# How much can we learn about missing data?: an exploration of a clinical trial in psychiatry

**DOI:** 10.1111/j.1467-985X.2009.00627.x

**Published:** 2010-07

**Authors:** Dan Jackson, Ian R White, Morven Leese

**Affiliations:** Medical Research Council Biostatistics UnitCambridge, UK; Institute of PsychiatryLondon, UK

**Keywords:** Informatively missing, Missing data, Missingness not at random, Prior elicitation, Proxy data, Repeated attempts, Sensitivity analysis

## Abstract

When a randomized controlled trial has missing outcome data, any analysis is based on untestable assumptions, e.g. that the data are missing at random, or less commonly on other assumptions about the missing data mechanism. Given such assumptions, there is an extensive literature on suitable methods of analysis. However, little is known about what assumptions are appropriate. We use two sources of ancillary data to explore the missing data mechanism in a trial of adherence therapy in patients with schizophrenia: carer-reported (proxy) outcomes and the number of contact attempts. This requires additional assumptions to be made whose plausibility we discuss. Proxy outcomes are found to be unhelpful in this trial because they are insufficiently associated with patient outcome and because the ancillary assumptions are implausible. The number of attempts required to achieve a follow-up interview is helpful and suggests that these data are unlikely to depart far from being missing at random. We also perform sensitivity analyses to departures from missingness at random, based on the investigators’ prior beliefs elicited at the start of the trial. Wider use of techniques such as these will help to inform the choice of suitable assumptions for the analysis of randomized controlled trials.

## 1. Introduction

Missing data in any research project are a cause for concern, but despite investigators’ best efforts they are often unavoidable. This paper focuses on missing outcomes in randomized clinical trials, but similar issues would arise in randomized and non-randomized experiments in all areas of research. Missing outcomes have two effects: they reduce precision and power, and they may introduce bias. There is little that the statistician can do about loss of precision, except to make best use of the data that are available—e.g. to be sure not to exclude from the analysis individuals who dropped out before the end of the study but who nevertheless reported intermediate values of the outcome (Wood *et al.*, 2004). However, the statistician can aim to reduce bias through suitable choice of an analysis.

All statistical analyses with missing data make assumptions, some of which explicitly specify the values of the missing data: e.g. that missing values are failures, as in smoking cessation trials (Sutton and Gilbert, 2007). Other assumptions make implicit assumptions about the similarity of distributions, such as ‘last observation carried forward’. It is usually better to make assumptions about the missing data mechanism, defined as the probability of missing data given the observed and unobserved data (Little, 1995). A widely used classification is missingness completely at random, where the probability of missing data does not depend on observed or unobserved data, missingness at random (MAR), where the probability of missing data does not depend on the unobserved data, conditional on the observed data, and missingness not at random (MNAR) where the probability of missing data does depend on the unobserved data, conditional on the observed data (Little and Rubin, 2002). Little (1995) reviewed several classes of missing data mechanism including covariate-dependent missingness completely at random and random-effect-dependent missingness at random. If the data are missing at random and the parameter spaces of the distributions for the outcomes and the selection process are distinct then the missing data mechanism is said to be ignorable, and analyses that observe the likelihood principle may avoid modelling the missing data mechanism when making inferences concerning the outcome data parameters (Little and Rubin, 2002).

An MAR assumption is widely proposed as a starting point for analysis, since it makes full use of the available data and is computationally reasonably straightforward and stable (Molenberghs *et al.*, 2004; Carpenter and Kenward, 2008). However, the MAR assumption is rarely plausible, and it is important to consider alternatives. These might take the form of excluding particular terms from the missing data mechanism: e.g. assuming that missingness is independent of past outcomes given the current outcome (Brown, 1990; Michiels and Molenberghs, 1997). Alternatively, one might assume a more general missing data model and assign particular values to the coefficient(s) of the current outcome in a sensitivity analysis (Rotnitzky *et al.*, 1998; Kenward *et al.*, 2001) or place a prior distribution on these parameters (Forster and Smith, 1998; Scharfstein *et al.*, 2003). Estimating a general MNAR missing data model has been proposed (Diggle and Kenward, 1994) but is highly dependent on distributional assumptions (Little, 1995; Kenward, 1998).

Thus the key issue in making a suitable choice of analysis is to decide what assumptions are plausible in a particular data set. This is a challenging task. Subject matter knowledge is crucial, but the literature tends to focus on exploring previously measured predictors of non-response (see for example Gray *et al.* (1996) in a survey setting), not on the more difficult but more important task of exploring the role of the outcome itself in the missing data mechanism.

It is, however, possible to explore the missing data mechanism more fully. One key idea is to quantify the difficulty of obtaining outcome data by the number of contact attempts (e.g. mailings of a questionnaire or telephone calls) and to assume that individuals who did not respond at all are more similar to those who were difficult to contact than to those who were easy to contact. This is often used informally (e.g. Kypri *et al.* (2004)) and is known as the ‘continuum of resistance’ model in the survey literature, although it is not universally accepted (Lin and Schaeffer, 1995). Alho (1990) formalized the idea statistically by using a model relating response at each contact attempt to the true outcome and other variables, which is identified by the assumption that the coefficients in this model are the same across contact attempts (though the intercept need not be). This approach has been used to estimate an informative missing data mechanism in a survey of Gulf war veterans (Wood *et al.*, 2006). Another idea is to exploit proxy (auxiliary) outcomes, such as a report by a carer. These data are usually used to make the MAR assumption more plausible (Ibrahim *et al.*, 2001; Huang *et al.*, 2005), but they could also be used in combination with other assumptions about the missing data mechanism.

This paper uses data from the QUATRO trial (Gray *et al.*, 2006) to assess the extent to which one can explore the missing data mechanism given rich data. This trial evaluated the effect of adherence therapy of self-reported quality of life of people with severe mental illness. The QUATRO investigators were particularly concerned by the possibility of bias due to missing data and therefore obtained three extra sources of data: they quantified their prior beliefs about the differences between observed and missing data at the start of the trial, they asked carers for their views about the patient's quality of life and they recorded the number of patient interviews that were arranged before one was successfully completed. Our main focus is on what we can learn about the missing data mechanism in this trial and on whether this sort of richer data could valuably be collected in other trials, but we also study the extent to which the conclusions of the QUATRO trial are affected by making different plausible assumptions about the missing data. Although previous case-studies have explored the use of prior beliefs (White *et al.*, 2007) and the number of contact attempts (Wood *et al.*, 2006), this is the first case-study to compare these methods critically and to include proxy responses.

The paper is arranged as follows. The QUATRO trial is described in Section 2 and the process for eliciting prior beliefs concerning the nature of the missing data is described in Section 3. The QUATRO trial is reanalysed in Section 4, initially assuming that the data are missing at random but then with a sensitivity analysis, informed by the elicited priors, to examine the robustness of inferences to this assumption. Analyses using the elicited priors more directly are also performed in Section 4. The use of carers’ proxy scores is considered in Section 5 but this proves unhelpful, as the proxy scores are too poorly correlated with the actual final health scores of participants. In Section 6, data concerning the number of contact attempts are used: this additional information proves helpful and suggests that the data are unlikely to depart far from being missing at random. A discussion in Section 7 concludes the paper.

## 2. The QUATRO complete-case analysis

The QUATRO trial (Gray *et al.*, 2006) was a single-blind, multicentre randomized controlled trial of the effectiveness of adherence therapy for participants with schizophrenia. The trial included 409 participants in four centres: Amsterdam (the Netherlands), Leipzig (Germany), London (England) and Verona (Italy). Participants were recruited from June 2002 to October 2003 from people under the care of mental health services and were individually randomized to receive eight sessions of adherence therapy (intervention) or health education (control) where the control allows for therapist time and relationship. The primary *a priori* hypothesis was that adherence therapy would result in an improved quality of life for people with schizophrenia, compared with health education. The interventions were delivered in routine general adult mental health settings. The inclusion and exclusion criteria were described in detail by Gray *et al.* (2006). Assessments were undertaken at baseline and at 52 weeks’ follow-up.

Attention here will focus on the trial's primary outcome, participants’ quality of life, self-reported via the SF-36 survey (Ware, 1993) and summarized via the mental health component score MCS where a higher MCS-score implies a better quality of life. MCS had sample mean 39 and 41 at baseline and follow-up, and standard deviation (pooled across the four centres) 11 and 12 respectively.

The main trial analysis was a complete-case analysis, excluding individuals with missing data at baseline or follow-up; the centre and randomized group are known for every participant. All analyses were completed on an intention-to-treat basis. The final quality-of-life score was regressed on randomized group, adjusted for the baseline score and centre. This gave an estimated intervention effect of −0.40 (intervention minus control) with a 95% confidence interval of (−2.56, 1.76); negative values correspond to a harmful effect of intervention (Gray *et al.* 2006). This was sufficient to exclude the difference of 6 points (equivalent to a medium standard effect size; Gray *et al.* (2006)) which was prespecified in the power calculation, and the trial was therefore reported as providing evidence for the lack of effect of adherence therapy. These results do not allow for the missing data (although sensitivity analyses did do so).

Only 349 of the 409 participants have both their baseline and their final quality-of-life scores recorded. Table 1 summarizes the pattern of missing data by intervention group. Missing values at baseline are not unknown in psychiatry, especially in self-completed questionnaires. This is because the participant may be registered in the trial but unable to complete some or all baseline and/or follow-up questionnaires within the measurement ‘window’. There is, however, a relatively small amount of missing data at baseline (10 and 13 participants have missing baselines in the intervention and control groups respectively) compared with final scores (29 and 13 participants respectively) and hence the potential for bias is largely due to the imbalance and larger amount of missing data at the end of the trial; missing data at baseline are not a source of bias (White and Thompson, 2005). Although the difference in proportions of participants providing final scores recorded is not statistically significant, it does suggest that the more demanding adherence therapy might result in a greater risk of participants failing, for whatever specific reason, to complete the interview process. Combining this with the natural concern that failure to complete the trial might be associated with a poor final health score, these issues raise the concern that the complete-case analysis of the QUATRO trial might exaggerate the intervention effect. The five participants who provided neither baseline nor final scores are retained in the analyses that follow as they contribute to MNAR analyses.

**Table 1:** Pattern of missingness for the QUATRO trial

*MCS outcomes recorded*	*Intervention*	*Control*	*Total*
Baseline only	26	11	37
Final only	10	8	18
Neither outcome	3	2	5
Both outcomes	165	184	349
Total	204	205	409

## 3. Eliciting prior beliefs

Because of concern about possible bias due to missing outcomes, the investigators’ prior beliefs about differences between the observed and unobserved data were elicited. These beliefs were elicited during the data collection but were not used in the original data analysis (Gray *et al.*, 2006). Elicitation of priors has been much discussed in general (see O'Hagan *et al.* (2006) for a summary) but rarely carried out in medical applications. We used a questionnaire based on one by Parmar *et al.* (1994) which we have previously used in printed form to elicit beliefs about informative missingness in another case-study (White *et al.*, 2007).

Training of experts followed by face-to-face elicitation is the ideal (O'Hagan *et al.*, 2006) but was impractical on this occasion, and instead a spreadsheet was prepared and e-mailed to investigators in the four centres. Training would provide the opportunity to prevent common misconceptions, e.g. that the questionnaire refers to the mean score within groups, rather than individuals, and to explain the meaning of any terms that might cause confusion. As some of the resulting elicited correlations are 0 and 1, as explained below, encouraging experts to think more carefully about questions that are used to obtain these would seem to be especially valuable. We do not propose our analyses in Section 4.4, which use elicited priors directly, be taken as primary but it is of interest to see how the elicited information affects the inferences from our model. Further questions could also be asked about missing observations at baseline and questionnaires conducted by telephone interview, rather than using a spreadsheet, might be preferable provided that due care was taken to ensure that all the experts’ beliefs were elicited in exactly the same manner.

The spreadsheet was completed individually by three investigators from London, and collectively at each of the other three centres; we refer to these informants as ‘experts’. All centres responded to the elicitation questionnaire and hence there is no missing information but the way in which the London centre conducted this exercise differs from the other three.

The elicitation tool first asked the following question, with regard to the intervention group:

‘Suppose the mean MCS of those who respond to the final questionnaire is 40 with standard deviation 10 (so that about 95% of these responders have values between 20 and 60). What is your expectation for the mean MCS for those who do not respond to the final questionnaire, compared with those who did respond?’

**Fig. 1:**
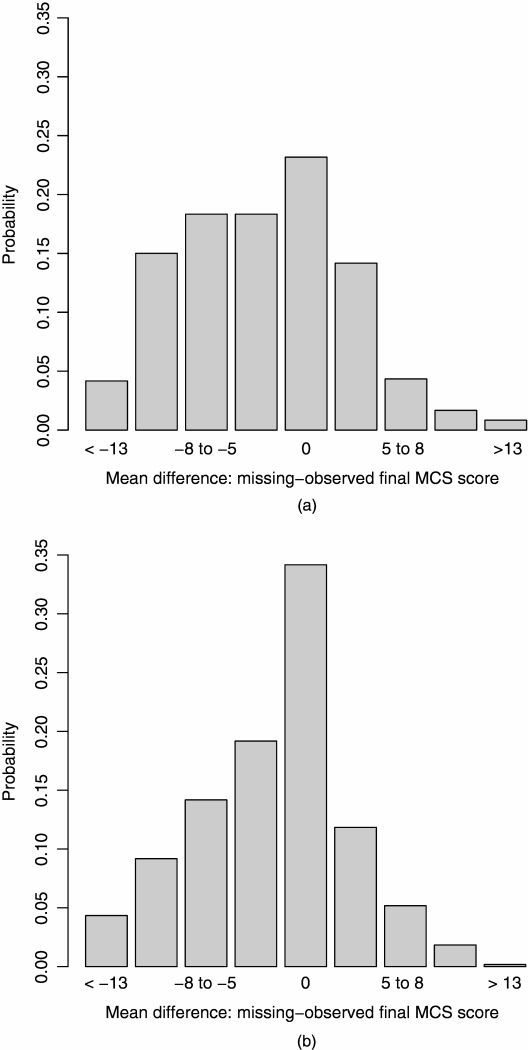
Expert opinion relating missing and observed quality of life in (a) the intervention and (b) the control groups

The experts were asked to distribute a total weight of 100 across nine categories: lower than responders by 1–4, 5–8, 9–12 or 13 or more points; the same as responders; or higher than responders by 1–4, 5–8, 9–12 or 13 or more points. The results of different experts were similar, so they were averaged (a linear opinion pooling rule; Genest and Zidek (1986)) giving the pooled beliefs that are shown in Fig. 1(a). A similar question was asked about the control group, leading to the results in Fig. 1(b). Using the category midpoints and 14.5 for the most extreme categories, the mean of this distribution of the difference between missing and observed scores in the intervention group is −2.9 with standard deviation 5.7; in the control this is −2.1 with standard deviation 5.2.

The two distributions in Fig. 1 both indicate an expert belief that missing health scores are likely (but not certain) to be less than those that are observed. There appears to be stronger belief that this is so in the intervention group.

Our analysis requires the prior correlation between the differences in the two arms: a measure of how closely the experts’ beliefs about the two arms were related. To assess this, during the elicitation process the experts’ own *average difference* between missing and observed scores *in the intervention group* was computed, using the representative values for each category, and their *maximum difference in the control group* was also noted. Experts were then asked

‘If I told you the non-responder/responder difference in the control arm really was as large as [their maximum value], what would be your best guess for the non-responder/responder difference in the adherence therapy arm? Would it still be [their average difference in the adherence therapy arm] or would it change to [their maximum difference in the adherence therapy arm] or somewhere in between?’

Experts who did not change their intervention group opinion are interpreted as having uncorrelated beliefs about the two arms and those who changed their beliefs to their maximum difference are interpreted as having perfectly positively correlated beliefs. Answers between these two extremes were interpreted as providing a positive correlation, obtained by linear interpolation. This process resulted in expert correlations of 0, 0, 0, 0.29, 0.73 and 1. The inconsistency between these values means that it is very difficult to reflect the beliefs about the experts’ perceived similarity between two arms by using a single prior distribution, so two contrasting possibilities are examined in Section 4.4.

## 4. A reanalysis of the QUATRO trial taking into account the missing data

In this section we set up a model for the QUATRO trial data, perform an analysis where the data are assumed to be missing at random and explore the effect of departures from this. The model will be extended in subsequent sections to incorporate proxy and ease-of-contact data.

**Fig. 2:**
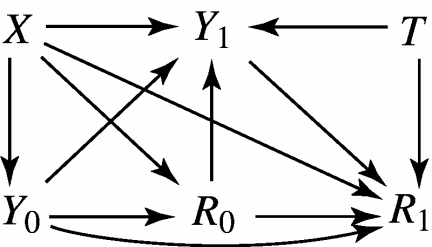
Directed acyclic graph of the model used for the QUATRO data

We model the data with the help of the directed acyclic graph in Fig. 2, modelling variables in the order *X* (centre), *Y*_0_ (baseline MCS-score), *R*_0_ (an indicator for observing *Y*_0_), *T* (an indicator random variable for randomization into the intervention group), *Y*_1_ (follow-up MCS-score) and *R*_1_ (an indicator for observing *Y*_1_). Each variable is modelled conditionally on all ‘previous’ variables in the list, with the exception of randomization, *T*, which is assumed independent of the previous variables. Since the models for *R*_0_ and *R*_1_ depend on the unobserved scores (and the mean of *Y*_1_ depends on *R*_0_), the model allows the data to be missing not at random.

The baseline scores were modelled by using a normal distribution with marginal mean linear in *X*, 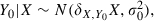 using an intercept and three dummy variables for the four centres that comprise *X*. We use *δ*_*A*,*B*_ to denote the coefficient of *A* in the model for *B*. Conditional on *X* and *Y*_0_, *R*_0_ was modelled by using the logistic regression 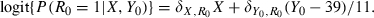


Note that *Y*_0_ is entered into the model by subtracting the sample mean of the observed scores and then dividing by the pooled standard deviation; this is to avoid numerical difficulties when implementing the Markov chain Monte Carlo algorithm and to make the resulting regression coefficients more interpretable. The coefficient in the logistic regression of *R*_0_ on *Y*_0_ represents the increase in log-odds-ratio of reporting *Y*_0_ associated with an increase in *Y*_0_ of about 1 standard deviation. Note that it is the joint model assumed for *Y*_0_ and *Y*_1_ which provides information concerning this coefficient as, on their own, data on *Y*_0_ and *R*_0_ give no information about the relationship between them. We model *Y*_1_ and then *R*_1_ by using normal and logistic regression models conditional on all previous variables in the list 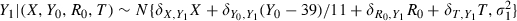 where *δ*_*T*,*Y*_1__ is the treatment effect and parameter of central interest, and 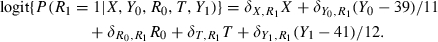
(1)

For some models (such as models F and G in Table 2), we wish to allow different *δ*_*Y*_1_,*R*_1__ in the two intervention arms. This is implemented by replacing *δ*_*Y*_1_,*R*_1__ in equation (1) with *δ*_(*Y*_1_,*R*_1_,T)_*T*+*δ*_(*Y*_1_,*R*_1_,C)_(1−*T*), where *δ*_(*Y*_1_,*R*_1_,T)_ and *δ*_(*Y*_1_,*R*_1_,C)_ denote the parameter *δ*_*Y*_1_,*R*_1__ in the intervention and control groups respectively.

**Table 2:** Results from the sensitivity analysis†

*Model*	*δ*_*Y*_0_,*R*_0__	*δ*_*Y*_1_,*R*_1__	Δ*(Y*_1,T_*)*	Δ*(Y*_1,C_*)*	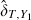
A	0	0	−0.01 (2.12)	0.12 (3.04)	−0.33 (1.08)
B	0.5	0	−0.01 (2.12)	0.14 (3.04)	−0.33 (1.09)
C	1	0	−0.02 (2.12)	0.11 (3.06)	−0.33 (1.08)
D	0	0.5	−4.49 (2.13)	−4.51 (3.07)	−0.65 (1.10)
E	0	1	−8.69 (2.16)	−9.04 (3.08)	−0.95 (1.11)
F	0	0‡ 1§	−0.05 (2.14)	−8.88 (3.02)	0.25 (1.10)
G	0	1‡ 0§	−8.49 (2.12)	0.35 (3.11)	−1.53 (1.10)

† Δ(*Y*_1,T_) and Δ(*Y*_1,C_) represent the expectation of the posterior distribution of the difference between the means of the missing and observed *Y*_1_ in the intervention and control groups respectively, and 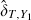 denotes the intervention effect. Standard deviations are in parentheses.

‡ In the intervention group.

§ In the control group.

This model involves quite a large number of parameters and missing observations would require to be integrated out of the likelihood, so the direct maximization of the resulting likelihood is a non-trivial task. Partly because of this difficulty, and also because we intend to make use of the prior distributions elicited from experts, Bayesian analyses were performed throughout, using Monte Carlo Markov chains produced by WinBUGS (Lunn *et al.*, 2000), and the means of the resulting posterior distributions were used as estimates. Unless stated otherwise, uniform priors over sufficiently large ranges to cover the region where the likelihood is not negligible were used for all *δ*-parameters, and standard uninformative gamma(0.001,0.001) distributions were used for the priors of the precisions of *Y*_0_ and *Y*_1_ (i.e. the reciprocals of the variances 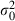 and 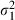). A burn-in of 25000 iterations for each of four chains was used for all analyses and a further 25000 simulations (providing 100000 simulations across the four chains) were used to make inferences. The traces of all simulated variables were carefully examined to verify convergence of the chains (all traces left little ‘white space’) and the WinBUGS implementation of the Gelman–Rubin convergence statistic, as modified by Brooks and Gelman (1998), was also examined. For all variables, both the pooled and the within-parameter variances were stable and the Gelman–Rubin statistics were close to 1 in every instance.

We shall explore various MNAR models in which missing data mechanisms are described in different ways and by different parameters. To compare results, we shall study the quantity Δ(*Y*_1,T_), which is defined as the posterior mean of the missing final score values minus the mean of the observed values in the intervention arm, and its counterpart Δ(*Y*_1,C_) in the control arm. These quantities measure departures from missingness completely at random, not from MAR, but we shall first evaluate them under an MAR model and pay attention to departures from their values under MAR.

It is in principle possible to estimate the model that was defined above without constraints, but any results are likely to be very dependent on distributional assumptions (Little, 1995; Kenward, 1998). In fact, our attempts to fit the full model without constraints, and with uninformative priors, were unsuccessful. This is because the model is very poorly identified, the resulting joint posterior distribution is very diffuse and numerical difficulties abound. Further analyses therefore make assumptions about some of the *δ*-parameters. A very large data set would provide more information, however, and avoid some of the difficulties that are encountered here.

### 4.1. An analysis assuming that the data are missing at random

**Fig. 3:**
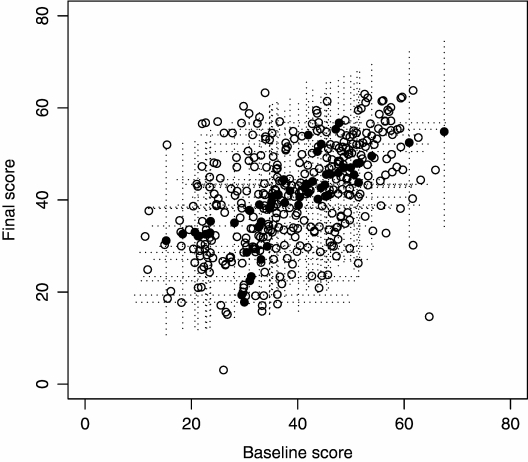
Plot of final score against baseline score, assuming that unobserved data are missing at random: ○, participants where both scores are observed; •, participants where one of these scores has been replaced by the posterior mean; ⋮, …., 95% posterior credible intervals for the imputed scores

One way to perform an analysis under the assumption that both the baseline and the final scores are missing at random is to assume that the scores (*Y*_0_,*Y*_1_) are conditionally independent of the indicators (*R*_0_,*R*_1_), by constraining *δ*_*Y*_0_,*R*_0__=*δ*_*Y*_0_,*R*_1__=*δ*_*R*_0_,*Y*_1__=*δ*_*Y*_1_,*R*_1__=0. This gives a point estimate of the intervention effect of 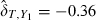, with a standard deviation of 1.08, which is in good agreement with the complete-case analysis that was described above. By monitoring the missing *Y*_0_ and *Y*_1_ when running the Markov chain Monte Carlo algorithm, their distribution can be obtained and the implications of the model for the missing values can be assessed. Under the assumption that the data are missing at random, the expectation of the posterior distribution of the difference between the means of the missing and observed *Y*_1_ in the intervention is just Δ(*Y*_1,T_)=0.20 with a posterior standard deviation of 2.1. In the control this difference is Δ(*Y*_1,C_)=0.54 with standard deviation of 3.0. These very small differences, compared with the sample standard deviation of *Y*_1_ of 12, indicate that the assumption of data missing at random implies that the missing and observed final scores are very similar. Fig. 3 also shows the implications of this assumption, where *Y*_1_ is plotted against *Y*_0_. Here the open points represent participants where both observations are observed, and full points denote values where either or both of *Y*_0_ and *Y*_1_ are not observed but have been replaced by the mean from their posterior distribution; the lines indicate 95% posterior credible intervals for these unobserved scores. Fig. 3 further demonstrates that the assumption that these data are missing at random implies that missing observations are similar to those that have been observed.

### 4.2. A sensitivity analysis

To explore the effect of departures from MAR, we performed a sensitivity analysis by constraining the sensitivity parameters *δ*_*Y*_0_,*R*_0__ and *δ*_*Y*_1_,*R*_1__ to a range of particular values as informed by the elicited prior beliefs. The expert opinion that was described in Section 3 relates directly to the difference between the means of observed and missing final scores, but here this dependence is modelled by using the logistic regression for *R*_1_ through *δ*_*Y*_1_,*R*_1__, a value which is to be constrained in the sensitivity analysis.

The results of Chene and Thompson (1996) allow us to relate the parameters in a pattern mixture model, for normally distributed data, to the informatively missing parameter in the corresponding selection model. If *σ*^2^ denotes the variance of *Y* (which is assumed the same in both patterns) and *μ*_o_ and *μ*_m_ denote the mean for cases (observed; *R*=1) and controls (missing; *R*=0) then the log-odds of being a case conditional on *y* is 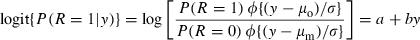 where *φ*(·) denotes the standard normal density function and *b*=(*μ*_o_−*μ*_m_)/*σ*^2^. Noting that standardized scores were used as covariates in the logistic regressions, standardized differences between the means of the two treatment groups are approximately equal to values of *δ*_*Y*_1_,*R*_1__. Since the vast majority of the elicited distributions for the difference between the means lie within the interval [−10,10], and the expert opinion elicited and shown in Fig. 1 was obtained by assuming a standard deviation of 10, we take *δ*_*Y*_1_,*R*_1__ to be unlikely to lie outside the interval [−1,1]. By considering only scenarios where good health scores are more likely to be reported, the primary concern that was raised in Section 2, this further restricts the range to [0,1]. This same interval is used for the corresponding missing baseline model parameter *δ*_*Y*_0_,*R*_0__ in the sensitivity analysis, and for the corresponding parameter for the logistic regression for the proxy scores in Section 5, as the baseline and proxy scores are likely to be missing for similar reasons to those for *Y*_1_.

We start with the choice *δ*_*Y*_0_,*R*_0__=*δ*_*Y*_1_,*R*_1__=0, which is conceptually close to MAR but is not MAR because it allows associations between *R*_1_ and *Y*_0_ when *Y*_0_ may be missing, and *R*_0_ and *Y*_1_ when *Y*_1_ may be missing. This (model A) and six further possible pairs of values for *δ*_*Y*_0_,*R*_0__ and *δ*_*Y*_1_,*R*_1__ are examined in Table 2. Initially *δ*_*Y*_1_,*R*_1__ is assumed to be 0 and *δ*_*Y*_0_,*R*_0__ is allowed to vary between 0 and 1 (models B and C). The model for the probability of reporting the baseline health score in models A–C does not appreciably affect the conclusions, and hence *δ*_*Y*_0_,*R*_0__ will be set to 0 in all the models that follow. In particular, model A provides very similar results to the MAR model, as expected.

**Fig. 4:**
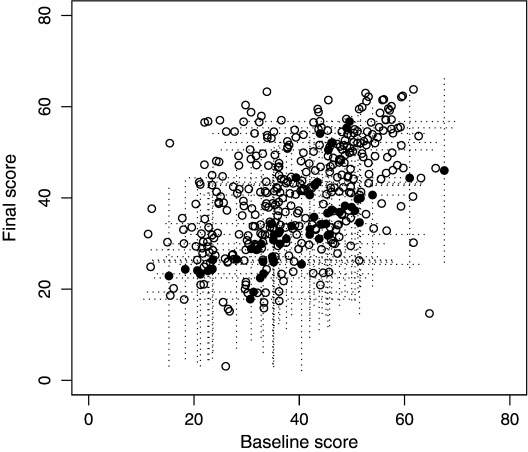
Plot of final score against baseline score, assuming model E in Table 2: ○, participants where both scores are observed; •, participants where one of these scores has been replaced by the posterior mean; ⋮, …., 95% posterior credible intervals for the imputed scores

As *δ*_*Y*_1_,*R*_1__ moves away from 0 and towards 1 (models D and E) the implications of this model for the intervention effect become more severe. Assuming *δ*_*Y*_1_,*R*_1__=1 provides an expected difference between the means of the missing and observed final health scores of around Δ(*Y*_1,T_)≈Δ(*Y*_1,C_)≈−9 and an estimated intervention effect of 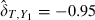, but with a similar standard deviation to that obtained when assuming that the data are missing at random. Fig. 4 is an analogous plot to Fig. 3 but shows the posterior distributions of the missing scores for model E, which represents quite an extreme case. Despite this, model E does not appear implausible in the light of Fig. 4, as the unobserved data are reasonably consistent with the observed data; the distributions of the missing *Y*_1_ are shifted down as a result of the large *δ*_*Y*_1_,*R*_1__ but are not inconsistent with the rest of the data. Models F and G further allow the *δ*_*Y*_0_,*R*_0__ to take the values 0 and 1 in the intervention and control, and then vice versa, and consider ‘worst case scenarios’. Perhaps most notably, model G provides an estimated intervention effect of around −1.5 with a standard deviation of around 1.1. There is no evidence that the intervention effect is not zero even in this extreme case, which is perhaps one of the most important conclusions for the QUATRO data. WinBUGS code for fitting model E in Table 2 is provided in Appendix A and can be modified to fit all the various models used.

### 4.3. Using a *t*-distribution to model the final scores

Kenward (1998) discussed another example with missing data, where outliers are influential. He found that the conclusions concerning the missing data mechanism are not robust to replacing the normal distribution with a *t*-distribution. Some of our analyses were therefore repeated using a *t*-distribution for *Y*_1_, with 10 and 5 degrees of freedom. As the degrees of freedom fell, very slightly smaller estimates of the intervention effect resulted (by around 0.06 when using 10 degrees of freedom and by a further 0.08 when using just 5). The conclusions from the above sensitivity analysis are insensitive to the introduction of a distribution for *Y*_1_ with heavy tails.

### 4.4. Using the elicited priors directly

We also fitted the model by using the elicited priors for *δ*_*Y*_1_,*R*_1__ more directly, assuming that the baseline scores are missing at random, so that *δ*_*Y*_0_,*R*_0__=0, and initially assuming that the prior beliefs for the intervention and control group are identical. Hence we have just a single parameter *δ*_*Y*_1_,*R*_1__ for both the intervention and the control groups and so we combine the distributions in Fig. 1 and further assuming normality gives a prior distribution of approximately *μ*_m_−*μ*_o_∼*N*(−2.5,30) for the difference between the means of missing and observed *Y*_1_; as noted above, the means of the distributions that are shown in Fig. 1 are −2.9 and −2.1 in the intervention and control groups respectively, with corresponding standard deviations of 5.7 and 5.2 and averaging these values gives the mean and standard deviation that were used for the prior.

Since this was elicited under the assumption that the standard deviation is 10, if the standard deviation is instead *σ* then this is interpreted as providing prior beliefs of *μ*_m_−*μ*_o_∼*N*(−0.25*σ*,0.3*σ*^2^), as experts are regarded as providing their beliefs in relation to *σ*. Following the argument that was provided by Chene and Thompson (1996), so that (*μ*_o_−*μ*_m_)/*σ*≈*δ*_*Y*_1_,*R*_1__, this roughly corresponds to a prior of *δ*_*Y*_1_,*R*_1__∼*N*(0.25,0.3). Normal distributions are used here for relative simplicity, although by using Markov chain Monte Carlo sampling other possibilities for representing the experts’ prior beliefs are also easily adopted. Using this prior, and with the same uninformative priors for the other parameters as before, resulted in a posterior distribution for *δ*_*Y*_1_,*R*_1__ which closely resembled the prior, appearing normally distributed with a posterior mean of 0.30 and a posterior standard deviation of 0.61. This results in an estimated intervention effect of −0.51 with a standard deviation of 1.16.

If instead two separate *δ*_*Y*_1_,*R*_1__ are used for the intervention and control groups, as in models F and G in Table 2, applying the argument of Chene and Thompson (1996) to Figs 1(a) and 1(b) separately gives approximate prior distributions of *N*(0.29,0.3) and *N*(0.21,0.3) for the intervention and control *δ*_*Y*_1_,*R*_1__ respectively. Assuming that these priors are independent, posteriors that resemble the prior normal distributions are obtained with posterior means of 0.30 and 0.26, and standard posterior deviations of 0.48 and 0.52. This results in an estimated intervention effect of −0.54 with a standard deviation of 1.23. Owing to the absence of information concerning the nature of the missing data, these analyses essentially return prior distributions of *δ*_*Y*_1_,*R*_1__ as posteriors and provide estimated intervention effects between those given by the sensitivity analyses with *δ*_*Y*_1_,*R*_1__=0 and *δ*_*Y*_1_,*R*_1__=0.5. Although not described in further detail here, this was also found when adding proxy scores to the model as described below. Perhaps the most important finding here is that these Bayesian analyses barely change the estimated intervention effect; the imbalance in the reporting of the final scores and the difference between the experts’ beliefs across the two treatment arms are both sufficiently small to ensure that these analyses provide fairly similar inferences to analyses that assume that data are missing at random.

## 5. Using carer's proxy health scores

To lessen the effect of the missing final MCS-scores, the carers’ assessments of the four key aspects of mental health that contribute to MCS (vitality, social functioning, role emotional and mental health) were recorded on a visual analogue scale. For simplicity, these were summed to give a proxy measure of the patient's quality of life, although this is not on the same scale as the patient-reported outcome (mean 20; standard deviation 6). Once again, there were missing observations: 379 of the 409 participants have their proxy outcomes recorded. However, 19 of the 42 participants with missing final MCS-scores have their proxy score recorded, so now only 23 participants have no recorded indication of their mental health at the conclusion of the trial.

Typically proxy outcomes have been used with the assumption that unobserved scores, given the observed proxy values, are missing at random (Ibrahim *et al.*, 2001). This is often sensible as the proxy scores should give a good indication of the true scores, and so the missing data mechanism can plausibly be assumed to depend on only the observed proxy scores. The assumption that missing final scores are missing at random need not be made here and seems implausible, as the empirical correlation between complete-case final and proxy scores is only 0.31. A straightforward extension of the directed acyclic graph that is shown in Fig. 2 can be used as a modelling framework where the nodes *Z* and *R*_*Z*_ are added to denote the proxy scores and an indicator variable for the proxy score being reported respectively.

Specifically, *Z* is modelled by using a normal linear regression model with mean linear in all variables shown in Fig. 2, and *R*_*Z*_ is modelled by using a logistic regression on these same variables and *Z*, where the three health scores were standardized in these two additional regressions in the same manner as above, and where both these additional regressions exclude interactions.

### 5.1. Analyses assuming missingness at random and a sensitivity analysis

On including the proxies in this way, an MAR model is obtained by ensuring that the scores (*Y*_0_,*Y*_1_,*Z*) and the indicators (*R*_0_,*R*_1_,*R*_*Z*_) are independent in a similar manner to the MAR model in Section 4. This gives an estimated intervention effect of 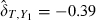 with a standard deviation of 1.09, so introducing the proxy scores under this assumption does not appreciably change the inferences that are made.

Constraining *δ*_*Y*_0_,*R*_0__, *δ*_*Y*_1_,*R*_1__ and *δ*_*Z*,*R*_*Z*__ to specific values made the model identifiable and so these were held fixed in a further sensitivity analysis. Table 3 shows the results where baseline values are assumed missing at random i.e. *δ*_*Y*_0_,*R*_0__=0. Models A–C suggest that the estimate of the intervention effect is also insensitive to the value of *δ*_*Z*,*R*_*Z*__ so this was held fixed at 0 in models D–G. Tables 2 and 3 suggest that the consequences of varying the parameter *δ*_*Y*_1_,*R*_1__ are similar irrespective of whether or not proxy values are used and that the introduction of these proxies under MAR adds little for this particular example.

**Table 3:** Results from the sensitivity analysis including the proxy scores†

*Model*	*δ*_*Y*_1_,*R*_1__	*δ*_*Z*,*R*_*Z*__	Δ*(Y*_1,T_*)*	Δ*(Y*_1,C_*)*	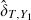
A	0	0	0.08 (2.10)	−0.03 (3.01)	−0.30 (1.09)
B	0	0.5	0.13 (2.11)	−0.08 (3.00)	−0.29 (1.08)
C	0	1	0.12 (2.11)	−0.09 (3.02)	−0.29 (1.09)
D	0.5	0	−4.40 (2.11)	−4.66 (3.03)	−0.63 (1.09)
E	1	0	−8.61 (2.14)	−9.15 (3.05)	−0.93 (1.11)
F	0‡ 1§	0	−0.05 (2.13)	−8.75 (3.00)	0.25 (1.09)
G	1‡ 0§	0	−8.27 (2.10)	−0.09 (3.04)	−1.46 (1.10)

† Baseline scores are assumed missing at random, i.e. *δ*_*Y*_0_,*R*_0__=0. Δ(*Y*_1,T_) and Δ(*Y*_1,C_) represent the expectation of the posterior distribution of the difference between the means of the missing and observed *Y*_1_ in the intervention and control groups respectively, and 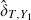 denotes the intervention effect. Standard deviations are in parentheses.

‡ In the intervention group.

§ In the control group.

### 5.2. A new assumption and a further sensitivity analysis

We now consider what other conditional independence assumptions about the proxy data are plausible. One possible assumption, as noted above, is that *Y*_1_ is missing at random once we condition on *Z*. A possible alternative is to assume that *R*_1_ remains conditionally associated with *Y*_1_ and *R*_*Z*_ remains conditionally associated with *Z*, but that *R*_1_ and *Z* are conditionally independent (*δ*_*R*_1_,*Z*_=0), i.e., given all the other variables in the model for *Z*, the carer's assessment of the patient's quality of life is independent of whether the patient responds. This is at first sight plausible, and probably would be in studies of physical health. In mental health work, however, it seems more likely that a patient's tendency to respond (rather than their actual behaviour on this particular occasion) would influence the carer's assessment of their quality of life, because carers are likely to regard engagement with society as beneficial. Despite this, the specific act of providing *Y*_1_ might plausibly be unassociated with *Z*, given all the other dependences in the model, and we shall assume for the moment that *δ*_*R*_1_,*Z*_=0 to illustrate how this type of analysis proceeds.

This places an alternative restriction on the role of *R*_1_ in the model and was found, on constraining *δ*_*R*_0_,*Y*_0__=*δ*_*Z*,*R*_*Z*__=0 as before, to make the model identifiable despite using uninformative priors on the remaining parameters. A point estimate of 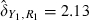 was obtained, with an estimated intervention effect of 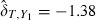, with a standard deviation of 1.19. The estimate of *δ*_*Y*_1_,*R*_1__ is much larger than the values that were explored in the sensitivity analysis, and considered plausible by expert opinion, and has resulted in a more harmful intervention effect than the model where *δ*_*Y*_1_,*R*_1__=1. This is because the observed association between *R*_1_ and *Z* drives the analysis; the small correlation between *Y*_1_ and *Z* (the empirical value is 0.31, using complete cases, as noted above), and assuming *δ*_*R*_1_,*Z*_=0 leaves only the association between *R*_1_ and *Y*_1_ to explain any association between the three variables *R*_1_, *Y*_1_ and *Z*. The parameter 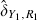 is therefore estimated to be large, with serious consequences for the estimate of the intervention effect.

Alternatives to *δ*_*R*_1_,*Z*_=0 were also considered. Constraining *δ*_*R*_1_,*Z*_=1, and noting that the pooled sample standard deviation of *Z* is 6 so that the effect of reporting *Y*_1_ directly alters the mean of *Z* by only around a sixth of a standard deviation, gives an estimate of 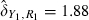, and an estimated intervention effect of 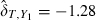, with a standard deviation of 1.19. Larger values of *δ*_*R*_1_,*Z*_ were also considered but for such values the estimation failed. This is because, as *δ*_*R*_1_,*Z*_ becomes larger, a very widely dispersed posterior distribution for *δ*_*Y*_1_,*R*_1__ results. The small change in *δ*_*R*_1_,*Z*_ from 0 to 1 is sufficient, however, to show that quite a marked change in key estimates occurs for relatively small changes in *δ*_*R*_1_,*Z*_ and hence constraining this, although facilitating the estimation of the parameter *δ*_*Y*_1_,*R*_1__ in the context of the sensitivity analysis, does not provide a particularly satisfactory solution for this particular example.

Other conditional independence assumptions could also be made, including for example assuming that *δ*_*Y*_1_,*R*_*Z*__=0. However, estimating the key parameter *δ*_*Y*_1_,*R*_1__, subject to conditional independence assumptions concerning the proxy values, does not seem satisfactory when proxies are so weakly correlated with final MCS-scores. Empirical associations between variables must be explained somewhere in the fitted model and constraining some dependences to 0 must affect other parts of the model; any serious implications for the key parameter *δ*_*Y*_1_,*R*_1__ have potentially direct and serious implications for the intervention effect. For examples where proxy scores are more strongly correlated with actual scores, such assumptions, and in particular the assumption that missing actual scores are missing at random given the proxy values, are more plausible.

## 6. Using information concerning the repeated attempts to contact participants

The interviewers in QUATRO were asked to record the process leading up to the final interview (or failure to interview) for each participant. The data that were collected included the date of each intended interview, how the interview was arranged (by agreement over the phone, or by letter or other indirect method) and the outcome of the intended interview (refused, not attended, attended but not completed or completed). Patients had up to nine interview attempts, but 42% had only one attempt and only 7% had more than three. The mean reported final health score by number of attempts made to contact participants (one, two, three or more than three) are shown in Table 4, where also the number of participants in each category is shown in parentheses. This suggests that participants who require further attempts to contact and attend interview tend to have lower health scores and by implication that participants with low health scores are less likely to attend interview at all. However, this trend is not statistically significant; regressing final MCS-score on the number of attempts for the participants reporting a final MCS-score results in a two-sided *p*-value of 0.14.

**Table 4:** Mean final MCS by number of contact attempts and trial arm†

*Number of attempts*	*Mean MCS for intervention group*	*Mean MCS for control group*
1	40.7 (87)	42.4 (83)
2	40.2 (96)	41.3 (96)
3	38.6 (9)	38.7 (10)
More than 3	36.2 (12)	36.8 (16)

† The numbers of participants in each category are in parentheses.

In this section we construct and estimate an MNAR model relating success at each attempt to the true (possibly unobserved) MCS and other fully observed covariates. For now the use of the proxy scores is dropped. Baseline scores are assumed to be missing at random, i.e. *δ*_*R*_0_,*Y*_0__=0.

To use these additional data, the model for *δ*_*Y*_1_,*R*_1__ (model 1) is replaced by a logistic regression for the *m*th attempt at contact being successful: 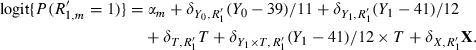
(2)

Here 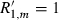 if the *m*th attempt is successful and 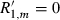 otherwise; *m*=1,2,3,…,9 denotes the attempt number, and the vector **X** denotes further covariates, including type of attempt (whether or not there was a verbal agreement to interview), three dummy variables to model the centre effects and possibly additional effects and interactions. Note that the notation in equation (2) emphasizes that the probability that the *m*th attempt is successful is assumed to depend on *m* only through the intercepts *α*_*m*_; for example the absence of *m* in the term 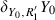 indicates that the same coefficient for *Y*_0_ is used in this logistic regression for all *m*, which is the crucial identifying assumption.

This model includes a trial arm by final score interaction, as the potential implications of such a term are obvious from Tables 2 and 3. The term 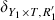 is constrained to 0 for models where no such interaction is desired. Because of the multiple attempts at contacting participants, the informatively missing parameter pertaining to *Y*_1_ in this regression becomes identifiable (Alho, 1990; Wood *et al.*, 2006). Very wide uniform prior distributions were used for the repeated attempts logistic regression variables and model (2) was built into the WinBUGS model, as described in Appendix A.

To choose the covariates **X** that were required to describe the data well, a standard complete-case logistic regression was initially fitted as a base model using the data involving just the first three attempts, including as covariates the attempt number, type of attempt, trial arm, centre and baseline and final MCS-scores. Then each of the possible first-order (two-factor) interaction terms were added in turn to this base model and any interactions that were significant at the 0.01-level were added to **X**; this rather stringent criterion was adopted as a relatively simple model was desired. Only the attempt number by centre interaction was significant at this level; this interaction was only extended as far as the third attempt, however, as very few attempts were a fourth or further attempt as noted above.

The key informatively missing parameters are the logistic regression coefficients for the effect of the standardized final score 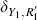 and, when included, 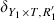. The intervention effect is still *δ*_*T*,*Y*_1__. Assuming that 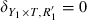 results in model A in Table 5, and allowing this interaction to be estimated results in model B. Although neither analysis provides convincing evidence that the final score directly influences the probability that the attempt is successful, the estimates point towards participants with better MCS-scores being more likely to attend interview and to report their MCS. This tendency is stronger in the intervention group, so estimates of intervention effect are slightly less than those from analyses that assume that all data are missing at random.

**Table 5:** Results from the models using the information concerning the repeated attempts to contact participants†

*Model*	Δ*(Y*_1,T_*)*	Δ*(Y*_1,C_*)*	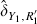	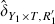	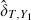
A	−1.54 (2.39)	−1.04 (3.13)	0.22 (0.17)	—	−0.46 (1.09)
B	−2.57 (2.60)	−0.60 (3.20)	0.14 (0.19)	0.25 (0.29)	−0.64 (1.11)
C	−2.64 (2.52)	−0.94 (3.16)	0.15 (0.19)	0.28 (0.28)	−0.61 (1.11)

† Baseline scores are assumed missing at random. Δ(*Y*_1,T_) and Δ(*Y*_1,C_) represent the expectation of the posterior distribution of the difference between the means of the missing and observed *Y*_1_ in the intervention and control groups respectively, and 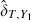 denotes the intervention effect. Standard deviations are in parentheses.

Finally, we combine models for the proxy outcomes and the repeated attempts. This uses the same model for the proxy outcomes, by simply adding the models for *Z* and *R*_*Z*_ in the same manner as before, but the *δ*_*R*_1_,*Y*_1__ arc is replaced by the logistic model for the repeated attempts. Fitting the same model as in model B but including the proxies with the assumptions that *δ*_*Y*_0_,*R*_0__=*δ*_*Z*,*R*_*Z*__=0 provides the results that are denoted by model C in Table 5. Once again introducing the proxy health scores in this way does little to change the conclusions. It is also worth noting that, if complementary log–log-link functions were used, instead of logits, in the various logistic regressions fitted, then the regression parameters in the repeated attempts regression (2) for 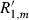 correspond to those in the model for *R*_1_. This is not so for the logistic regression models that were fitted here, but these are expected to have similar properties. In particular, the estimates of 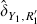 and 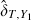 in Table 5 are consistent with the corresponding values from the sensitivity analyses shown in Tables 2 and 3, and the analyses in Section 4.4 that use the priors directly.

## 7. Discussion

The QUATRO trial provided rich data: proxy carers’ scores, information on the number of attempts at contacting participants and expert prior beliefs about the nature of the missing data. We set out to allow for MNAR in the trial analysis and to learn about the magnitude of possible departures from MAR. There is no information concerning the repeated attempts that were made to obtain baseline data but if this were available then this might also be useful as the logistic model for *R*_0_ that was described above could be replaced by a similar model for the repeated attempts. The model could also be extended to incorporate proxy scores at baseline for examples where these are available. Another possible way to extend the analysis is to use data on adherence to randomized allocation (in QUATRO, the number of sessions attended) to make the MAR assumption more plausible.

Using the carer's scores as proxies was not successful. They make MAR more plausible, but given their weak association with the true outcomes they were not very useful. The alternative model with proxy scores conditionally independent of the event that the final score was recorded was implausible and gave implausible results, but it might have been useful in a non-mental health trial, such as Ibrahim *et al.* (2001). It is perhaps interesting to note that the collection of the proxies in QUATRO actually arose from discussion of the prior between the investigators, showing the importance of statistical engagement in trial design.

Using the information on the number of attempts to contact participants was more successful. The results for the informatively missing parameters were reasonably precise and excluded large departures from MAR and large differences in MAR parameters between arms; the latter is most important in generating bias. However, this model also makes assumptions to make it identifiable; in particular, the effect of possibly missing outcomes on the probability of response is assumed constant across contact attempts. With three or more follow-up visits, the assumptions are partly testable (Wood *et al.*, 2006). Molenberghs *et al.* (2009) have established the impossibility of discriminating between MNAR and MAR mechanisms by using the evidence provided by data so we know that the assumptions made by the repeated attempts model are crucial when attempting to ascertain the potential departure from the MAR model that was developed here.

All models that were considered in the sensitivity analyses are well identified. The sample size is relatively large and so the posterior distributions for the treatment effect are well approximated by the usual asymptotic normal approximations. The resulting inferences for the treatment effect from the sensitivity analyses are therefore fairly robust to the precise form of the priors and give similar numerical results to analogous likelihood-based frequentist analyses. For the analyses in Section 4.4, which use the elicited priors directly, there is a stronger case for the examination of alternative prior distributions for other parameters in the model which more accurately reflect clinicians’ opinion.

With specific regard to the QUATRO trial, the analyses that take missingness into account make the estimated effect of the intervention slightly more harmful but there is still no evidence of an intervention effect on any analysis that was considered, and credible intervals always exclude the clinically significant value of 6 points. The results from fitting the repeated attempts model support, but do not prove, the MAR assumption for these data and indicate that the range of values that was used in the sensitivity analysis was more than adequate.

The sorts of exercises that are suggested here should be more widely attempted. They can be used to assess the plausibility of assumptions such as MAR and to explore the sensitivity of trials’ findings to departures from MAR. In the longer term, researchers should aim to amass wider experience of evidence about missing data mechanisms in different areas of medical research, to inform future analyses of randomized controlled trials.

## Appendix A: WinBUGS code

Here we give WinBUGS code for fitting model E in Table 2 to the 409 QUATRO participants. ‘x1’, ‘x2’ and ‘x3’ are indicator vectors (vectors of length 409 containing 0s and 1s) for membership of the second, third and fourth centres respectively. ‘R0’ and ‘R1’ are indicator vectors for the event that the baseline and final scores are observed respectively and ‘treat’ and ‘rtreat’ are indicator vectors for the event that participants are in the intervention (treatment) and control group respectively. The vectors ‘Y0’ and ‘Y1’ are the baseline and final scores respectively.

model

{

n<− 409# 409 QUATRO participants

# Priors:

# Flat priors for all logistic regression parameters alpha, except the two

# parameters (alpha[5] and alpha[13]) that are constrained to 0 and 1 in model E:

alpha[1]∼ dunif(−20,20)

…

alpha[5]<−0

…

alpha[12]∼ dunif(−20,20)

alpha[13]<− 1

# Flat priors for all linear regression parameter beta; however, note that

# beta[1] and beta[5] are the model intercepts and require priors centred # at around 40:

beta[1]∼ dunif(20,60)

beta[2]∼dunif(−20,20)

…

beta[5] ∼dunif(20,60)

…

beta[11]∼ dunif(−20,20)

# The parameter beta[11] is the treatment effect.

# Uninformative priors for the precisions:

prec0∼ dgamma(0.001,0.001)

prec1∼ dgamma(0.001,0.001)

# The likelihood:

for(i in 1:n) {

# Likelihood for the scores:

mu0[i]<−beta[1]+beta[2]* x1[i]+beta[3]* x2[i]+beta[4]* x3[i]

Y0[i] ∼ dnorm(mu0[i],prec0)

mu1[i]<−beta[5]+beta[6] * x1[i]+beta[7] * x2[i]+beta[8]* x3[i]+beta[9]* (Y0[i]−39)/11+beta[10]* R0[i]+beta[11]* treat[i]

Y1[i] ∼dnorm(mu1[i],prec1)

# Likelihood for R_0_ and R_1_:

logit(pbaseline[i])<− alpha[1]+alpha[2] * x1[i]+alpha[3]* x4[i]+alpha[4]* x5[i]+alpha[5] * (Y0[i]−39)/11

R0[i] ∼ dbern(pbaseline[i])

logit(pfinal[i])<− alpha[6]+alpha[7] * x1[i]+alpha[8] * x2[i]+alpha[9] * x3[i]+alpha[10] * (Y0[i]−39)/11+alpha[11]* R0[i]+alpha[12]* treat[i] +alpha[13]*(Y1[i]−41)/12

R1[i] ∼ dbern(pfinal[i])

}

# sum[1] and sum[2] below are the sums of the control and intervention final

# scores. Simulated posterior values are used for missing values when # computing these sums.

sum[1]<−inprod(Y1[],rtreat[])

sum[2]<− inprod(Y2[],treat[])

}

### A.1. Repeated attempts

When modelling the repeated attempts data by using model (2), the logistic regression for the final score in the above code is removed and is replaced by

for(j in 1:759) {

#759 attempts were made to contact QUATRO participants

resp[j]∼dbern(p.resp[j])

logit(p.resp[j])<−intercept[attempt[j]]+gamma[1] * (Y[id[j],1]−39)/11+

gamma[2] * (Y[id[j],2]−40)/11+gamma[3] * treat[id[j]]+gamma[4] * treat[id[j]] * (Y[id[j],2]−40)/11

}

Here ‘id’ and ‘attempt’ are vectors of length 759 providing data for each of the attempts: id takes values 1–409 and gives the participant involved in each attempt; attempt gives the attempt number. The further covariates **X** in model (2) can be added as required and flat priors are placed on the intercept and gamma parameters.
